# Physiological and Pathological Significance of Esophageal TRP Channels: Special Focus on TRPV4 in Esophageal Epithelial Cells

**DOI:** 10.3390/ijms23094550

**Published:** 2022-04-20

**Authors:** Ammar Boudaka, Makoto Tominaga

**Affiliations:** 1 Department of Physiology, College of Medicine and Health Sciences, Sultan Qaboos University, Al-Khoud, P.O. Box 35, Muscat 123, Oman; 2 Division of Cell Signaling, National Institute for Physiological Sciences, Okazaki 444-8787, Aichi, Japan; tominaga@nips.ac.jp; 3 Department of Physiological Sciences, SOKENDAI (The Graduate University for Advanced Studies), Okazaki 444-8787, Aichi, Japan; 4 Exploratory Research Center on Life and Living Systems, Thermal Biology Group, Okazaki 444-8787, Aichi, Japan

**Keywords:** ATP, Ca^2+^ homeostasis, esophageal cancer, esophageal epithelial cells, esophagus, GERD, ion transporters, mechanosensation, TRPV4, wound healing

## Abstract

Transient receptor potential vanilloid 4 (TRPV4) is a non-selective cation channel that is broadly expressed in different human tissues, including the digestive system, where it acts as a molecular sensor and a transducer that regulates a variety of functional activities. Despite the extensive research to determine the role of this channel in the physiology and pathophysiology of different organs, the unique morphological and functional features of TRPV4 in the esophagus remain largely unknown. Ten years ago, TRPV4 was shown to be highly expressed in esophageal epithelial cells where its activation induces Ca^2+^-dependent ATP release, which, in turn, mediates several functions, ranging from mechanosensation to wound healing. This review summarizes the research progress on TRPV4, and focuses on the functional expression of TRPV4 in esophageal epithelium and its possible role in different esophageal diseases that would support TRPV4 as a candidate target for future therapeutic approaches to treat patients with these conditions.

## 1. Introduction

The main functions of the alimentary canal are the propulsion of food, mechanical and chemical digestion, secretion of enzymes, absorption, and protection against different pathogens and toxins, as well as formation and expulsion of fecal material. These functions are carried out on a daily basis and their regulation depends on a proper set of functional signals from a variety of sources ranging from the luminal contents to the nervous system [1]. Contents of ingested meals interact directly with the alimentary canal epithelium and indirectly with two subsets of nerve fibers, intrinsic and extrinsic, that innervate the gastrointestinal tract. The local nervous system, also termed the enteric nervous system, exists as an intricate neuronal mesh enclosed in the wall of the alimentary canal and is involved in detecting chemical, mechanical, and osmolarity changes, as well as in regulating gut secretory and motility functions. Both limbs of the enteric nervous system, the submucosal and myenteric plexuses, are, in turn, modulated by extrinsic vagal and spinal efferent nerve fibers as part of a reflex arc initiated by stimulation of extrinsic afferent nerves originating from jugular, nodose, and dorsal root ganglia. Such an arrangement provides the alimentary canal with a complex and elaborate set of molecular sensors that scrutinize the luminal contents, discern adverse conditions in the digestive tract wall and lumen, and modulate secretory activity [2].

The esophagus is part of the alimentary canal that extends from the mouth to the anus. Like other segments of the canal, the esophagus wall is composed of the same four basic layers, but the histological features of most of these layers do differ. For example, the outermost layer is adventitia rather than serosa. The esophagus muscularis externa contains striated muscle fibers in its proximal region, and distally, these striated fibers mix with smooth muscle fibers in variable proportions in a species-dependent manner. The submucosa of the esophagus contains connective tissue cells, blood and lymphatic vessels, nerves, and submucosal glands. Interestingly, esophagus tissue in pigs and humans has large numbers of submucosal glands, which are absent in rodent esophagus [3]. Submucosal glands are tubuloacinar glands that are dispersed throughout the submucosa. They are lined with epithelial cells and mainly secrete mucus, bicarbonate, and growth factors, and their primary function is to lubricate the esophagus and to protect the tissue from the damaging effects of acidic gastric refluxate [4]. Another unique feature of the esophageal wall structure is its mucosa, in which the epithelium has a stratified squamous type with varying thickness and degree of keratinization, which is dependent on the species under investigation. In the esophagus of rodents, squamous epithelial cells are organized in four to five layers and are usually keratinized. Meanwhile, in the thicker esophageal mucosa of humans and pigs, the squamous cells are arranged in up to 10–15 layers, and the epithelial surface is not keratinized [3]. Sentient responses to chemical, thermal, and mechanical stimuli occur in the esophagus and ion transporters mediate many of these responses.

## 2. TRP Channels in the Esophageal Wall

Ion transporters play an important role in gastrointestinal physiology and pathophysiology. Several studies have been conducted to explore the physiological expression of different ion channels in the wall of the alimentary canal [5]. In the esophageal mucosa, epithelial cells, also known as esophageal keratinocytes, express a broad variety of receptors and ion channels such as calcium sensing receptors (CaSRs) [6], protease-activated receptor 2 (PAR2) [7], epithelial sodium channels (ENaCs) [8,9], acid-sensing ion channels (ASICs) [10], and members of the transient receptor potential channel family [11,12,13]. These channels and receptors work conjointly as multiple sensors for relevant chemical and physical stimuli.

Transient receptor potential (TRP) channels are non-selective cation channels that mediate influx of Ca^2+^, Mg^2+^ and monovalent cations in different cell types [14]. The first mammalian thermosensitive TRP channel was cloned from sensory neurons by Professor David Julius and his team in 1997. This TRP channel was first called vanilloid receptor subtype 1 (VR1; now named TRPV1). VR1 was shown to be a heat-sensing calcium-permeable ion channel that is stimulated by capsaicin (the substance that provides the piquancy of hot red chili peppers), noxious heat, and low pH, suggesting its possible role as a pain transducer [15]. This discovery opened the doors for subsequent discovery of other members of the TRP channel superfamily that make substantial contributions to different physiological and pathological conditions in almost every organ of the human body. Professor Julius was recently awarded the 2021 Nobel Prize in Physiology or Medicine, which he shared with the molecular biologist and neuroscientist Professor Ardem Patapoutian.

A functional TRP channel has a central cation-permeable hydrophilic pore encircled by four subunits, and each subunit has six transmembrane domains with cytoplasmic C and N termini [16]. To date, mammalian TRP channels are a large superfamily comprising 28 members that are categorized into six subfamilies based on their amino acid sequence: ankyrin (TRPA1), canonical (TRPC1-7), melastatin (TRPM1-8), mucolipin (TRPML1-3), polycystin (PC) (TRPP1-3), and vanilloid (TRPV1-6) [14,17,18]. Members of this family of ion channels are expressed in almost every cell in the body including the alimentary canal [13,19,20,21,22,23], where they play a pivotal role in the homeostasis and different diseases of the GI tract [24,25].

In the digestive tract, TRP channels are mainly expressed by primary afferent sensory neurons that emerge from enteric nervous system neurons, and vagal and dorsal root ganglia that jointly innervate the alimentary canal [25]. Nevertheless, several recent studies also revealed functional expression of TRP channels in non-neuronal cells of the gut such as esophageal epithelial cells, enterocytes, and enteroendocrine cells [12,26,27,28]. The TRPV1 channel, the most studied member of the TRP family, is extensively expressed in the alimentary canal, mostly by primary afferent sensory and enteric neurons, but it is also expressed by enteroendocrine cells and mucosal epithelial cells [29]. The TRPA1 channel is expressed by mucosal cells as well as ganglia and enteric primary neurons that project to the alimentary canal. The TRPA1 channel is considered as one of the most promising polymodal chemosensors in the gut as it can be stimulated by an array of dietary and noxious molecules, such as allicin and isothiocyanates, as well as by products of oxidative stress and exogenous irritants [25,30]. The TRPM5 and TRPM8 channels, two members of the melastatin subfamily, are widely expressed in the alimentary canal. The TRPM5 channel is expressed by lingual taste bud receptor cells and gut chemosensory cells [31,32], whereas the TRPM8 channel mostly localizes to primary afferent neurons where it functions as a cold chemosensor [33,34].

Different members of the TRP superfamily were shown to be expressed in the esophageal wall (Table 1). For instance, TRPA1-expressing vagal sensory neurons and afferent nerves were identified in guinea pig esophagus, where they mediate long-lasting mechanical hypersensitivity of vagal nodose and TRPV1-positive jugular afferent C fibers that is induced following mast cell activation [35]. Moreover, TRPA1 channel agonists preferentially stimulate vagal nodose nociceptive fibers, whereas jugular nerve fibers have a relatively weaker response to these agonists [36]. TRPA1-expressing vagal sensory neurons and afferent C-fiber subtypes are also sensitized by prolonged allergen challenge in guinea pig esophagus [37]. Although upregulation of mucosal TRPA1 channel expression was shown to mediate macroscopic and microscopic gastric mucosal injury in a rat model of gastritis [38], there is no current evidence to support a role for esophageal epithelial TRPA1 channel in mediating esophagitis or esophageal hypersensitivity in gastroesophageal reflux disease (GERD) patients.

Likewise, TRPM8-expressing vagal C fibers of jugular, but not nodose, origin express TRPM8 mRNA and respond to TRPM8 channel agonists as evidenced by results from both patch clamp and calcium imaging techniques [39]. This finding suggests a putative role for these vagal jugular C fibers in esophageal sensation and nociception. This possibility is further supported by the ability of menthol (TRPM8 agonist) infusion to elicit cold sensations in the esophagi of healthy subjects compared to the heartburn evoked in GERD patients [40].

The TRPV1 channel is expressed in esophageal sensory neurons and afferent nerve fibers of different animal species [13], and mediates capsaicin-induced heartburn and esophageal sensitivity [41]. Upregulation of TRPV1 channel expression in mucosal, including intraepithelial, sensory fibers might contribute to symptoms experienced by patients with GERD [47,48]. Moreover, TRPV1 channel overexpression, at both the mRNA and protein level, was shown in the esophageal mucosa of patients with non-erosive reflux disease (NERD) and erosive reflux disease (ERD), suggesting that TRPV1 channel might contribute to NERD symptoms and possibly explain the esophageal hypersensitivity exhibited by these patients [11]. These observations were further supported by a recent study that revealed significantly increased expression of TRPV1 channel on superficial mucosal sensory nerves in NERD patients and concomitant, exclusively increased expression of ASIC3 channel on epithelial cells from patients with NERD and ERD, indicating a sensory role for esophageal epithelial cells in acid reflux perception. These epithelial cells act interdependently with TRPV1-expressing mucosal nerves to augment esophageal hypersensitivity in patients with NERD [49]. This result further supports previous findings in a murine NERD model that indicate a role for mucosal TRPV1 channel overexpression in esophageal inflammation and acid-induced decrease of esophageal transepithelial electrical resistance [50]. TRPV1 channel expression is not restricted to sensory neurons as several recent studies have shown that it is expressed in esophageal epithelial cells. For instance, in human esophageal epithelial cells, the TRPV1 channel mediates interleukin 8 production and induces intracellular production of reactive oxygen species [42]. Furthermore, both in vivo and in vitro studies have shown TRPV1 channel expression on murine esophageal epithelial cells that was increased upon exposure to acid and then was reverted after exposure to menthol [43]. However, the TRPV1 channel does not seem to play a role in the esophageal mucosal barrier in patients with NERD [42]. On the other hand, the TRPV2 channel was shown to be expressed by nitrergic myenteric neurons in the mouse esophagus [44], suggesting that it could modulate esophageal motility via myenteric co-innervation of vagal efferent fibers innervating esophageal striated muscle fibers [21,22,23]. This possibility was further supported by the observed upregulation of TRPV2 channel in nitrergic myenteric inhibitory neurons of the lower esophageal sphincter (LES) in a rat model of reflux esophagitis. This augmented TRPV2 channel expression induces nitric oxide-mediated relaxation of LES resulting in acid reflux that could contribute to the development of GERD. Oral instillation of the TRPV2 channel antagonist tranilast in this esophagitis model significantly ameliorated body weight loss and improved epithelial thickness, as well as lessened severity of esophageal lesions [51]. Although TRPV2, TRPV3, TRPV4, and TRPV6 channels were shown to be present in the mucosa of mouse esophagus and cultured esophageal keratinocytes, the functional role of TRPV2, TRPV3, and TRPV6 channels in esophageal epithelial cells remains unclear [12,46]. High levels of TRPV6 channel expression in human and mouse esophageal stratified epithelia suggest a putative role for this ion channel in mediating cell survival and programmed cell death signaling pathways [46].

Members of the TRP channel superfamily were shown to play an important role in the progression and proliferation of esophageal cancer, especially esophageal squamous cell carcinoma (ESCC). The finding that dysregulation of TRPC6, TRPM2, TRPM7, TRPM8, TRPV1, TRPV2, and TRPV6 channel expression was linked to ESCC pathogenesis and prognosis suggests that these TRP family members could be used as prognostic markers and would be promising therapeutic targets [52]. For instance, the expression levels of TRPC6 mRNA and protein are higher in ESCC tissue compared to normal esophageal tissue, and the inhibition of TRPC6 channel activity in human ESCC cells suppresses cell proliferation and induces G2/M phase arrest, as well as decreases tumor formation in a mouse xenograft model [53]. Likewise, TRPM2 channel expression is increased in ESCC tumor tissue and is involved in calcium-mediated inhibition of cell proliferation and enhanced apoptosis of ESCC cells [54]. TRPM7 channel expression level was also reported to be a valuable prognostic factor in ESCC patients, and siRNA-based silencing of TRPM7 increases ESCC cell proliferation, migration, and invasion [55]. Meanwhile, mRNA and protein expression of another melastatin TRP channel, TRPM8, was shown to be upregulated in esophageal cancer cells compared to adjacent normal tissue. This finding suggests that the TRPM8 channel plays a crucial pro-tumor role in the pathogenesis of esophageal cancer and, thus, could be a therapeutic target [56]. TRPV1 channel expression was also shown to be upregulated in ESCC cells and TRPV1 channel overactivation promoted by recurrent stimulation with heat or capsaicin enhances cellular proliferation and migration of ESCC cells [57]. Similarly, the TRPV2 channel is overexpressed in ESCC cells and TRPV2 channel silencing suppresses ESCC cell proliferation and cell cycle progression, induces cell apoptosis, and is also associated with poor prognosis and low 5-year overall survival [58]. TRPV6 channel expression is significantly downregulated in human ESCC compared to adjacent nontumor tissues and this downregulation was correlated with advanced cancer stage and low survival rate [59].

**Figure 1:**
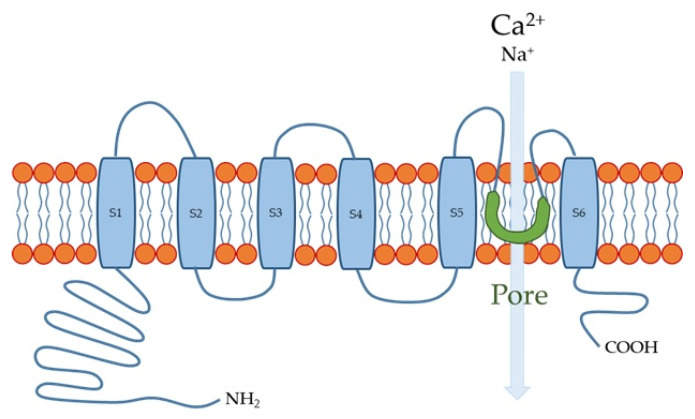
Transient receptor potential vanilloid 4 (TRPV4) channel structure. S1-S6 are six membrane-spanning helices.

The human TRPV4 channel has 871 amino acids with intracellular N- and C-termini and six transmembrane spanning (S1–S6) α-helices (Figure 1). S5, S6, and the interconnecting loop form the central cation-permeable pore [14,60]. The TRPV4 channel was initially reported to be an osmo- or mechano-sensor [61,62] that can be activated by moderate temperatures (>27 °C) [63] and UV light [64], as well as several endogenous and exogenous substances (Table 2). The TRPV4 channel is a non-selective cation channel that is widely expressed in many tissues throughout the body, where it plays important roles in several physiological functions [65,66,67]. In the esophagus, TRPV4 channel is expressed in the basal and intermediate layers of the esophageal epithelium [12,68].

Agents that tweak TRPV4 channel activity could be promising therapeutics for the treatment of many disease conditions including congestive heart failure, respiratory and gastrointestinal disorders, osteoarthritis, and pain [69]. For instance, due to the proposed damaging role of TRPV4 channel on the alveolar-capillary barrier and in the development of lung edema, inhibitors of TRPV4 channel activity could be used to protect or restore the damage to this barrier in patients with various respiratory diseases, including COVID-19 [70,71]. Research geared toward discovering TRPV4 channel modulators for therapeutic use began around ten years ago and has evolved significantly in the last few years. The TRPV4 channel has, indeed, proven to be a greatly druggable target. At least nine novel chemotypes have potential as templates for potent TRPV4 channel agonists or antagonists that have oral bioavailability and other drug-like properties. At least two TRPV4 channel antagonists have demonstrated sufficient properties and preclinical safety profiles to be recommended as drug candidates. To date, GSK2798745 is the only potent and selective TRPV4 channel inhibitor that has been investigated in four separate early phase clinical trials: a Phase 1 study to assess effects on alveolar-septal barrier permeability following LPS challenge in healthy subjects; a Phase 2a study in participants with chronic cough; a first-in-human trial in healthy participants and stable heart failure patients; and a Phase 2a trial in congestive heart failure patients. GSK2798745 was found to be safe and well-tolerated, and to exhibit some positive efficacy trends in patients with heart failure [72]. Meanwhile, the progress in developing TRPV4 channel agonists as medicines has lagged behind that for antagonists due to the toxicity caused by systemic activation of TRPV4 channel [69].

In this review, we summarize recent research progress about the functional expression of TRPV4 channel in esophageal epithelium, with a special focus on its possible role in different esophageal diseases and the potential of targeting this channel for the development of therapeutic approaches for these conditions.

## 3. TRPV4 in Mechanosensation

In addition to its role in providing a conduit for food from the pharynx to the stomach, the esophagus can function as a sensory organ due to its dual innervation by primary afferents having vagal and spinal origins that terminate either in the muscularis externa or en route to the mucosa, where they branch into a delicate mesh of fine varicose fibers [88]. Some mucosal afferents have intraepithelial extensions that place them in close proximity to esophageal luminal contents and may impart mechano-, thermo-, or chemosensory functions [22,89,90]. Chemical, mechanical, thermal, and noxious stimuli acting on the esophageal wall are transduced to action potentials, either directly or indirectly, by a multitude of receptors expressed on esophageal sensory nerves [88] or non-neuronal cells, such as esophageal keratinocytes [12]. These action potentials are then transmitted to the central nervous system via the spinal and vagal afferents. The most prominent terminal structures of vagal afferent fibers in the esophageal muscle coat are termed intraganglionic laminar endings (IGLEs) [91]. The polymodal vagal afferents mainly carry mechano-, chemo-, and thermosensations [88], but several studies were unable to identify the exact molecular mechanosensor that is expressed by the vagal afferents and confers these activities. Nevertheless, some of these studies did reveal that vagal afferents in rat and mouse esophagi express the ionotropic purinergic receptors P2X_2_ and P2X_3_ [92,93]. In recent years, adenosine triphosphate (ATP) has become widely recognized as a rapid synaptic transmitter in both peripheral and central divisions of the nervous system [94]. Several subsequent studies showed that many non-neuronal cells, including different epithelial cells, can release ATP in response to multiple stimuli including subjecting cell membranes to stretch [95,96]. In P2X_3_ knockout mice, mechanical distension induced ATP release from the esophagus, which was similar to that seen in wild-type (WT) mice, whereas activation of vagal afferents was reduced relative to WT mice [97]. Meanwhile, a P2X_3_ agonist was shown to stimulate mechanosensitive vagal afferents in mouse esophagus [98], suggesting that ATP release induced by mechanical stimuli and its action on P2X_3_ receptors plays an important role in esophageal mechanosensation. Considering the ability of skin keratinocytes to release ATP upon stimulation [99], we proposed that esophageal keratinocytes might have a similar capacity to release ATP in response to various stimuli (including stretch). The released ATP can, in turn, stimulate P2X_2_ and P2X_3_-expressing esophageal vagal afferents, which provides important clues about the missing pieces in the puzzle of molecular mechanotransduction in the esophagus.

**Figure 2:**
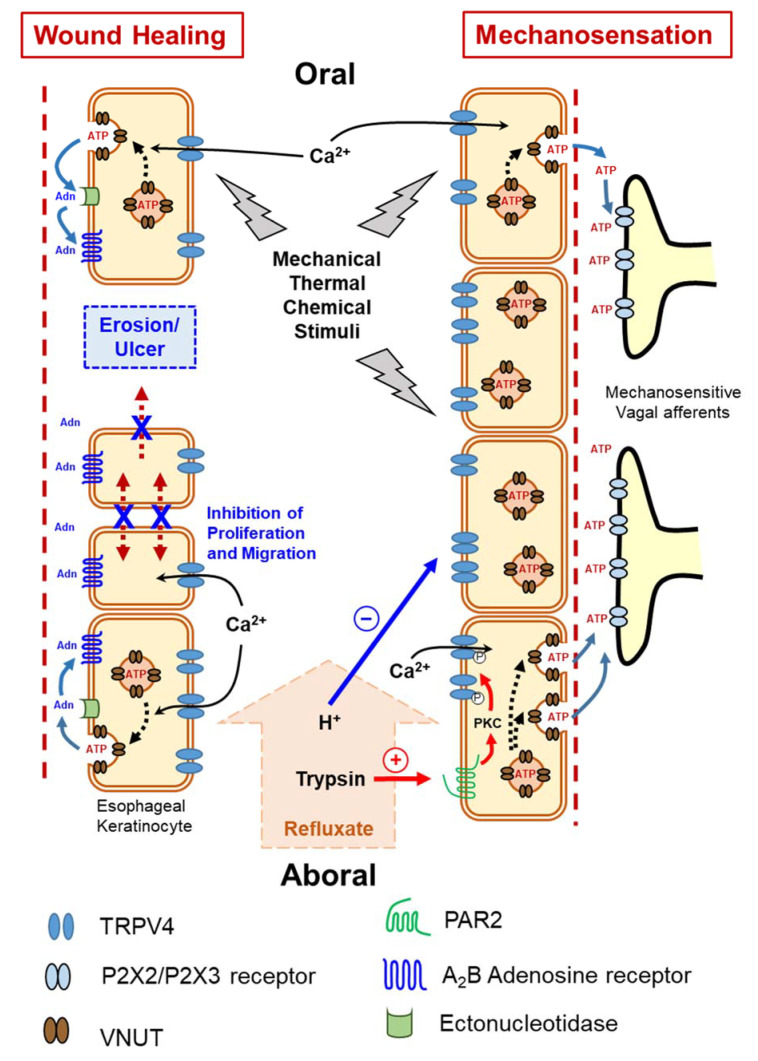
Functional expression of transient receptor potential vanilloid 4 (TRPV4) channel in esophageal keratinocytes and its possible role in mechanosensation and wound healing via Ca^2+^-dependent exocytotic ATP release. Adn = Adenosine, ATP = adenosine triphosphate, P2X2/P2X3 = subtypes of purinergic receptors, PAR2 = protease-activated receptor 2, VNUT = vesicular nucleotide transporter.

We explored whether the TRPV4 channel functions as a mechanosensor in the esophagus. We found that TRPV4 mRNA and proteins are expressed in esophageal keratinocytes harvested from WT mice. Using a patch-clamp technique, we showed that several known TRPV4 channel activators, including heat and the agonist GSK1016790A, evoked TRPV4-like currents in cultured esophageal keratinocytes from WT, but not *Trpv4* knockout (TRPV4-KO) mice. Moreover, these activators, as well as stretch, increased cytosolic Ca^2+^ concentrations in the cultured keratinocytes. Heat and the TRPV4 channel agonist GSK1016790A also significantly increased ATP release from cultured WT esophageal keratinocytes, but not from TRPV4-KO cells. The ability of esophageal keratinocytes to pack ATP into vesicles in preparation for release was supported by the finding that these cells express the newly identified vesicle ATP transporter, vesicular nucleotide transporter (VNUT), at both the mRNA and protein levels. Thus, our findings clearly support the hypothesis that TRPV4 channel mediates Ca^2+^-dependent exocytotic ATP release in response to mechanical, thermal and chemical stimuli. The released ATP, in turn, activates P2X-expressing vagal (IGLEs and mucosal) afferents [12]. This esophageal keratinocyte-vagal afferent crosstalk with TRPV4 channel acting as a possible epithelial mechanosensitive molecule could be a vital component of esophageal mechanotransduction (Figure 2). This observation was supported by our subsequent findings showing morphological and functional expression of TRPV4 channel in murine and rat gastric epithelia. In this recent study, TRPV4-expressing gastric epithelial cells responded to various TRPV4 channel stimulants through Ca^2+^-dependent ATP release that could contribute to gastric emptying, most likely by triggering a local reflex arc intrinsic to the stomach wall that involves ATP release mediated by P2X2 and P2X3-expressing putative gastric mechanosensing vagal afferent intraganglionic laminar endings located in close proximity to the epithelium [19].

## 4. TRPV4 in Cell Proliferation and Migration

Various luminal insults, such as gastric refluxate, could compromise esophageal epithelial integrity and possibly cause esophageal erosions and, in more severe cases, ulcerations. Calcium and heat are among many factors that affect epithelial wound healing [100,101]. As an ion transporter with high permeability to Ca^2+^ upon stimulation, TRPV4 channel attracted our attention as a candidate regulator of esophageal epithelial wound healing. We have shown that esophageal keratinocytes obtained from TRPV4-KO mice exhibit an augmented ability for in vitro wound healing involving both enhanced cell proliferation and migration that was slowed when the cells were transfected with TRPV4 cDNA. Moreover, mechanical stimuli in the form of cyclic tensile strain slowed wound healing to a greater degree in WT compared to TRPV4-KO esophageal keratinocytes. These results clearly demonstrate that deletion of TRPV4 enhances in vitro wound healing of cultured esophageal keratinocytes [26]. The ability of the TRPV4 channel to mediate Ca^2+^-dependent exocytotic release of ATP from cultured esophageal keratinocytes in response to mechanical, chemical, and thermal stimuli [12] raises the question of whether ATP, or one of its degradation products, plays a modulatory role in the observed repressive effect of TRPV4 channel on wound healing. Although exogenous ATP significantly slowed wound healing, the inability of apyrase (an ATP hydrolase) to affect gap closure or abolish the inhibitory effect of exogenous ATP ruled out a direct role for ATP in modulating the in vitro wound healing process, and suggests that ATP metabolites (e.g., ADP, AMP, and adenosine) are candidate modulators of wound healing [26]. Most extracellular adenosine is derived from the release and metabolism of adenine nucleotides, such as ATP, following diverse stimuli [102]. Ectonucleotidases are extracellular enzymes that metabolize released ATP to yield different products such as adenosine [103,104], which in turn acts on G-protein-coupled adenosine receptors to control several physiological processes, including cell proliferation [105]. Therefore, we hypothesized that the ATP metabolite adenosine could be a candidate molecule involved in modulating in vitro wound healing of esophageal keratinocytes. The observed ability of exogenous adenosine to delay wound healing further supports this possibility. This effect was shown to be mediated by the highly expressed A_2B_ adenosine receptor in esophageal mucosa and blocked by a selective A_2B_ adenosine receptor antagonist [26] (Figure 2).

Acid can also inhibit the acid-sensitive TRPV4 channel expressed by murine esophageal epithelial cells [68]. Collectively, the aforementioned findings suggest that protons in gastric refluxate could enhance wound healing through a TRPV4-suppressing effect and possibly act as a natural protective mechanism to withstand acid-induced injury.

## 5. TRPV4 in Esophageal Inflammation and Tumors

Gastroesophageal reflux disease (GERD) is a multi-factorial chronic disease that may involve esophageal inflammation associated with hypersensitivity to mechanical or heat stimuli as well as acids, and can be attributed to altered expression of different ion channels in the esophageal wall [3]. Based on the presence or absence of mucosal damage, GERD patients can be classified as having either erosive esophagitis (EE) or nonerosive reflux disorder (NERD) [106]. For instance, inflammation-mediated overexpression of the mucosal TRPV1 channel is thought to play a role in NERD and GERD [11,48]. Although there is no direct evidence of TRPV4 channel overexpression in the esophageal mucosa of NERD and GERD patients, Suzuki et al. showed that esophageal keratinocytes express PAR2 and TRPV4 mRNA and protein. They also showed PAR2 activation following exposure to trypsin upregulated TRPV4 channel function via the protein kinase C-mediated phosphorylation of TRPV4 serine residues. This TRPV4 channel phosphorylation increased ATP release in mouse esophageal keratinocytes [107]. The effects on esophageal vagal afferents conferred by enhanced ATP release could be responsible for the commonly observed mechanical hyperalgesia in NERD and GERD patients [108]. Thus, inhibition of TRPV4 channel by different antagonists (Table 2) could be a potential novel therapeutic strategy for symptomatic treatment of these conditions.

Eosinophilic esophagitis (EoE) is another chronic inflammatory disease that affects the esophageal mucosa and can be induced by food antigens [109]. Although recent studies reported that altered expression of at least two different ion transporters, anoctamin 1 (ANO1) and sodium-hydrogen exchanger member 3 (NHE3), could contribute to EoE pathogenesis [3], none of the mucosal TRP channels are thought to play a role in the condition and, thus, further investigation is needed to explore their possible role in EoE.

In Barrett’s esophagus, normal esophageal squamous epithelium is replaced by intestinal columnar cells [110]. This condition is an adaptation to the altered environment imposed by long-term GERD [110]. A change in the expression or function of different ion transporters has a significant role in the development of Barrett’s esophagus [3]. However, whether there is a clear role for esophageal epithelial TRPV4 channel in this pre-malignant metaplasia is unclear.

Like several other molecules, altered expression of the TRPV4 channel was observed to be closely related to tumor formation and metastasis. The TRPV4 channel was shown to be overexpressed in colorectal, lung, and gastric cancer cells relative to the respective healthy cells, but in prostate, skin, and esophageal cancer cells, TRPV4 channel expression was lower relative to healthy cells [111]. The overactivation of the TRPV4 channel associated with its overexpression in some tumors results in higher intracellular calcium, which, in turn, regulates downstream signaling pathways to affect the different tumorigenesis processes. Downregulation of the TRPV4 channel observed in other tumors might be ascribed to differences in the tumor microenvironment, which could affect tumor activity via alternate pathways. For instance, lack of expression of a given gene during cell maturation can inhibit differentiation processes, which are primarily responsible for tumorigenesis. The TRPV4 channel is highly expressed in healthy or inflamed epidermal epithelium that is similar to esophageal epithelium on a histological level, but is lower, or even absent, in precancerous lesions and non-melanoma skin cancers. The growth and differentiation of skin keratinocytes are affected by intracellular and extracellular Ca^2+^ concentrations. When extracellular Ca^2+^ concentrations are low, primary keratinocytes remain undifferentiated. In the presence of high Ca^2+^ concentrations, cell proliferation is suppressed and differentiation is, thus, facilitated [112]. In human skin keratinocytes, activation of the TRPV4 channel inhibits cell proliferation, induces apoptosis, and stimulates the release of IL8, which, in turn, downregulates TRPV4 channel expression [113,114]. Hypothetically, low expression of the TRPV4 channel in esophageal cancer cells decreases the release of ATP and, hence, reduces formation of adenosine. Low concentrations of intercellular adenosine, sensed by autocrine and paracrine communication between keratinocytes, will induce cell proliferation and migration [26]. In summary, the TRPV4 channel plays an important role in cell proliferation and differentiation that further affects cancer progression. These findings raise the possibility that pharmacological inhibition of the TRPV4 channel or a combination of TRPV4 channel antagonists (Table 1) with other chemotherapeutic agents might provide alternate treatment options for patients with esophageal cancer that have not responded to standard treatment. Thus, the potential of TRPV4 channel modulators warrants further investigation to explore a possible role for this channel in the diagnosis, treatment, and prognosis of esophageal tumors.

## 6. Conclusions

The TRPV4 channel is functionally expressed in esophageal epithelial cells, where it mediates Ca^2+^-dependent ATP release. The released ATP is directly involved in mechanotransduction and indirectly, via its metabolite adenosine, in regulation of esophageal cell proliferation and migration. These findings suggest that inhibition of TRPV4 channel might promote healing of esophageal erosions and ulcers, and provide treatment options for patients with mechanical hyperalgesia. Further studies are needed to explore the exact role of the TRPV4 channel and its downstream pathways in esophageal barrier integrity, submucosal gland secretion, NERD, GERD, Barrett’s esophagus, and esophageal tumors, since targeting this channel using currently available agonists and antagonists could provide promising therapeutic options for these conditions.

## Figures and Tables

**Table 1:** Expression of different TRP channels in the esophageal wall.

TRP Channel	Localization	Functional Role	Species	Reference
TRPA1	Vagal sensory neurons and afferent nerves	Mediate long-lasting mechanical hypersensitivity	Guinea pig	[35,36,37]
TRPM8	Jugular vagal C fibers	Esophageal sensation and nociception Cold sensation	Guinea pig Human	[39,40]
TRPV1	Sensory neurons and afferent nerve fibers	Mediates capsaicin-induced heartburn and esophageal sensitivity	Guinea pig, Human, Mouse, Rat	[13,41]
	Esophageal keratinocytes	Mediates IL8 production and induces intracellular production of reactive oxygen species	Human, Mouse	[42,43]
TRPV2	Nitrergic myenteric neurons	Possible modulation of esophageal motility via myenteric co-innervation of vagal efferent fibers	Mouse	[21,44]
TRPV4	Esophageal keratinocytes	Mediates mechanosensation via ATP release Delays in vitro wound healing by contributing to increases in levels of adenosine, derived from TRPV4-mediated ATP release	Mouse	[12,45]
TRPV6	Esophageal keratinocytes	Putative role in mediating cell survival and programmed cell death	Human, Mouse	[46]

NERD: nonerosive reflux disease; EE: erosive esophagitis.

**Table 2:** Transient receptor potential vanilloid 4 (TRPV4) channel agonists and antagonists.

Name	Selectivity	In Vivo/Route/Species	Reference
**Agonist**			
GSK1016790A	Non-selective	+(IV, SC) mice	[45,73]
4αPDD	Non- selective	In vivo	[45]
4αLPDD	Non- selective		[45]
4αPD	Non- selective		[74]
Phorbol 12 myristate 13-acetate	Non-selective		[45]
5,6-epoxyeicosatrienoic acids (5,6-EET)	Non-selective		[75,76,77]
Dimethylallyl pyrophosphate (DMAPP)	Non-selective	+(intraplantar) mice	[78]
Bisandrographolide A (BAA)	Non-selective		[74]
N-arachidonoyl taurine	Non-selective		[79]
Apigenin	No evidence		[80]
Cannabidivarin, Tetrahydrocannabidivarin	Non-selective		[45]
RN-1747	Non-selective		[81]
**Antagonist**			
HC-067047	Selective	+(SC) mice	[82,83]
Citral	Selective		[82]
RN-1734	Selective		[82]
GSK205	Selective	+(topical) mice	[82,84]
GSK2193874	Non-selective	+(IV, IP) mice and rats	[82,85]
Ruthenium red (RR)	Non-selective		[45,86,87]
Butamben	Non-selective		[82]
Capsazepine	Non-selective TRPV		[82]
Gd^3+^	Non-selective TRPV		[87]
La^3+^	Non-selective TRPV		[87]
